# Refining surgical models of osteoarthritis in mice and rats alters pain phenotype but not joint pathology

**DOI:** 10.1371/journal.pone.0239663

**Published:** 2020-09-29

**Authors:** Peter R. W. Gowler, Paul I. Mapp, James J. Burston, Mohsen Shahtaheri, David A. Walsh, Victoria Chapman

**Affiliations:** 1 Pain Centre Versus Arthritis, School of Life Sciences, Queen’s Medical Centre, University of Nottingham, Nottingham, United Kingdom; 2 Pain Centre Versus Arthritis, Academic Rheumatology, City Hospital, University of Nottingham, Nottingham, United Kingdom; University College London, UNITED KINGDOM

## Abstract

The relationship between osteoarthritis (OA) structural change and pain is complex. Surgical models of OA in rodents are often rapid in onset, limiting mechanistic utility and translational validity. We aimed to investigate the effect of refining surgical small rodent models of OA on both joint pathology and pain behaviour. Adult male C57BL/6 mice (n = 76, 10–11 weeks of age at time of surgery) underwent either traditional (transection of the medial meniscotibial ligament [MMTL]) or modified (MMTL left intact, transection of the coronary ligaments) DMM surgery, or sham surgery. Adult male Sprague Dawley rats (n = 76, weight 175-199g) underwent either modified meniscal transection (MMNX) surgery (transection of the medial meniscus whilst the medial collateral ligament is left intact) or sham surgery. Pain behaviours (weight bearing asymmetry [in mice and rats] and paw withdrawal thresholds [in rats]) were measured pre-surgery and weekly up to 16 weeks post-surgery. Post-mortem knee joints were scored for cartilage damage, synovitis, and osteophyte size. There was a significant increase in weight bearing asymmetry from 13 weeks following traditional, but not modified, DMM surgery when compared to sham operated mice. Both traditional and modified DMM surgery led to similar joint pathology. There was significant pain behaviour from 6 weeks following MMNX model compared to sham operated control rats. Synovitis was significant 4 weeks after MMNX surgery, whereas significant chondropathy was first evident 8 weeks post-surgery, compared to sham controls. Pain behaviour is not always present despite significant changes in medial tibial plateau cartilage damage and synovitis, reflecting the heterogeneity seen in human OA. The development of a slowly progressing surgical model of OA pain in the rat suggests that synovitis precedes pain behaviour and that chondropathy is evident later, providing the foundations for future mechanistic studies into the disease.

## Introduction

Osteoarthritis (OA) is a chronic disease affecting synovial joints, and pain is a significant symptom [1]. Difficulties in mapping pain phenotypes to structural changes in the joint have impeded the search for novel therapeutics for the treatment of joint pain. Clinically, OA is classified using radiographic methods which rely on the presence of osteophytes and joint space narrowing [2]. Although these measurements have been shown to be associated with joint pain [3], others have reported that these associations are weak [4]. Analysis by magnetic resonance imaging (MRI) has revealed that bone marrow lesions and synovitis are more strongly associated with OA pain [5, 6]. Thus, the contributions of structural pathology to joint pain remain unclear.

Small rodent models of OA are commonly used for pre-clinical research into the mechanisms that underlie chronic OA-induced joint pain [7]. These models aim to closely represent the pathology and physiology of the human disease in a reliable and reproducible manner. Destabilization of the medial meniscus (DMM) is commonly used as a surgical model of OA in the mouse [8]. In this model transection of the mediomeniscotibial ligament (MMTL) results in a slowly progressing joint pathology and pain phenotype [8, 9]. These changes occur from 4 weeks post-surgery with previous studies reporting severe chondropathy and pain behavior 12 weeks following surgery [9]. A slowly progressing model of OA, such as the DMM model, allows investigation of mechanisms which drive the onset of OA pain during the early timepoints.

Mice are a popular model species partly due to the relative ease with which their genome can be manipulated [10]. However, rats are in many respects a preferable species for various experimental pain research endpoints, and remain widely used [11]. A commonly used rat surgical model of OA is the medial meniscal transection (MNX) model, induced by the removal of the medial collateral ligament (MCL) and transection of the medial meniscus [12]. This surgery results in rapid joint degradation and pain behavior within 7 days [13]. To allow researchers more freedom in choosing the species that best suit their experimental question, a slow progressing surgical model of OA in the rat would have considerable utility. To date there is only limited evidence for slow progressing surgical models of OA in the rat [14, 15].

The aim of our study was to investigate how the onset of structural changes in the joint map onto pain behavioural phenotypes in rodent models of OA, and to develop a slowly progressing model in the rat. To achieve this, we varied the induction of the DMM model of OA and studied differences between joint pathology and pain on loading (as assessed by weight-bearing asymmetry) in the two models. We hypothesized that a more subtle induction of the DMM model would result in less pronounced joint pathology and would subsequently affect pain outcomes. We then modified the established MNX surgical method with the goal of inducing a slowly progressing surgical model of OA in the rat, to further study interactions between joint pathology and pain behaviour.

## Materials and methods

Experiments using animals were performed in accordance with the UK Animal (Scientific Procedures) Act (1986) and were approved by the University of Nottingham Animal Welfare and Ethical Review Body (AWERB). Animals were briefly anesthetised with 3% isoflurane carried by O_2_. At the end of the study animals were euthanised by an appropriate method (overdose of sodium pentobarbital) as laid out by Schedule 1 of the UK Animal (Scientific Procedures) Act (1986). These studies were performed in male mice and rats, and as such may not be generalisable to females. Adult male C57BL/6J mice (n = 76, 10–11 weeks of age at time of surgery, Charles River, Margate, UK) were housed in individually ventilated cages in temperature-controlled rooms under a 12-hour light dark cycle (7am-7pm). Adult male Sprague Dawley rats (n = 76, weight 175-199g, Charles River) were housed in conventional cages in temperature-controlled rooms under a 12-hour light dark cycle (7am– 7pm). All animals had access to standard rodent diet and water *ad libitum*. Experimenters were blinded to the treatment of the animals for the entire duration of the experiment, and animals were randomly allocated to groups by a third party.

### Behavioural testing

Prior to each testing session animals were habituated to the experimental room for at least 30 mins to help minimise stress. Behavioural testing was only carried out once the animals were calm and not showing signs of stress. Weight-bearing asymmetry was assessed using an incapacitance tester (Linton Instrumentations, Norfolk, UK) as previously described [16]. The weight-bearing protocol was the same for both rats and mice. Briefly, animals were placed in a Perspex box above the split weight sensors of the incapacitance meter. Measurements were taken once animals had settled into the correct position, hind-paws in the centre of each sensor, forepaws resting on the front of the box, and tail resting outside the slot at the back of the box. Each measurement takes the average weight placed on each sensor over a 3 second period, and 3 measurements were taken per session. Weight-bearing percentage was calculated as the difference in weight borne between the contralateral and ipsilateral hindlimbs divided by the total weight borne on both hindlimbs. Hind paw withdrawal thresholds were assessed using von Frey (VF) monofilaments in an up-down protocol as previously described [16, 17]. Briefly, rats were placed into testing chambers and allowed to settle before testing began. Once rats were calm and still VF hairs were gently applied to the plantar aspect of the hind-paws, starting with the 4g hair, for 3s. If there was no response to the hair, the next hair was applied to the hind-paw, waiting a minute between applications to prevent sensitization. The highest hair used for these studies was 26g to ensure that there was no tissue damage from the application of the VF hairs. Once a response, licking of the hind-paw or an exaggerated lifting of the hind-paw, was observed the next lowest hair was applied to ensure that there was no response to this stimulus. The lowest hair which elicited a response was taken as the absolute withdrawal threshold.

### Destabilisation of the medial meniscus in the mouse

During these studies we induced the DMM model of OA in mice through two variations of the surgery. For the traditional DMM experiment 18 male C57BL/6J mice aged 10–11 weeks of age at time of surgery were used. Following anaesthesia with isoflurane (3% carried by O_2_), mice underwent either destabilisation of the medial meniscus (n = 10) or sham surgery (n = 8) of the left knee joint. Before surgery, the knee was swabbed with Emla (eutectic mixture of local anaesthetics: 25mg/g lidocaine, 25mg/g prilocaine) cream. The surgery involved making an incision over the medial meniscus and then the knee joint capsule was blunt dissected open, the medial meniscotibial ligament (MMTL) was transected and the medial meniscus was destabilised. There is the potential that blunt dissection of the joint capsule could in itself cause significant damage to the joint and mediate some of the pain seen in these models, in order to mitigate this the sham surgery was the same as the DMM in all aspects except for the transection of the MMTL. Post-surgery animals were given access to mash to help with rehydration, and elongated waterspouts were added to drinking bottles to ensure animals did not need to reach up for access to water. Health monitoring, to assess signs of wound breakdown, limping, joint swelling, weight-loss, and general signs of distress was carried out twice a day for 7 days post-surgery, and then once a week for the duration of the study. This was the same procedure for all surgical experiment carried out in this study. If animals were found to show signs of these adverse events, then they were humanely euthanised by overdose of sodium pentobarbital. Note one mouse was euthanised at one-week post-surgery due to a breakdown of the wound. Following surgery pain behaviour was assessed by weight-bearing asymmetry once a week for 16 weeks. At the end of the study mice underwent transcardial perfusion with 4% paraformaldehyde (PFA) under terminal anaesthesia (overdose of sodium pentobarbital).

Thirty-four (34) male C57BL/6 mice aged 10–11 weeks of age at time of surgery were used to investigate the effects of the modified induction of the DMM surgeries (S1 Fig). Mice were anaesthetised with isoflurane (3% in O_2_) and then either underwent the modified destabilisation of the medial meniscus surgery (n = 16) or sham surgery (n = 18). The surgery involved making an incision over the medial meniscus before opening the joint capsule through blunt dissection. The coronary ligaments underneath the medial meniscus were then transected to destabilise the medial meniscus. The sham surgery was identical, but the coronary ligaments were not transected. Following surgery, weight-bearing asymmetry was measured once a week for 16 weeks. A separate cohort of 24 male C57BL/6J mice (n = 16 mDMM, n = 8 sham) were used to investigate the mDMM model over a longer time course, with weight-bearing asymmetry measured once a week for 20 weeks post-surgery. At the end of the study mice underwent transcardial perfusion with 4% paraformaldehyde (PFA) under terminal anaesthesia (overdose of sodium pentobarbital).

### Modified meniscus transection surgery in the rat

Seventy-six (76) adult male Sprague Dawley rats (175-199g) underwent either modified meniscal transection surgery (n = 38) or sham surgery (n = 38). Under isoflurane anaesthesia (3% in O_2_) an incision was made over the medial meniscus; the joint capsule was opened with a cautery torch to limit bleeding and the medial meniscus was transected close to the MMTL. Unlike the traditional MNX the medial collateral ligament was left intact. The sham surgery was completed following the blunt dissection of the joint capsule. Pain behaviour was measured in a subset of these rats (mMNX: n = 11, sham: n = 9) once a week for 16 weeks. Weight-bearing asymmetry and absolute hind-paw withdrawal thresholds were measured as previously described. At the end of the study rats underwent transcardial perfusion with 4% paraformaldehyde under terminal anaesthesia (overdose of sodium pentobarbital).

### Histological assessment of joint pathology

For the mouse models, knee joints were collected post-mortem 16 weeks post-surgery. The joints were fixed in 10% neutral buffered formalin for 48 hours before being decalcified in 10% ethylenediaminetetraacetic acid (EDTA) for 10 days. The joints were embedded in paraffin wax before being sectioned (5μm thick) coronally. These sections were then stained with haematoxylin and eosin (H&E). Three stained sections starting from the anterior portion of the joint when the tibia started to flatten and then at ~100μm intervals were taken from each knee joint per animal were then scored for medial tibial plateau chondropathy according to the following criteria; 0 –normal, 1- Small fibrillations without the loss of surface cartilage, 2- vertical clefts down the layer immediately below the superficial layer and some loss of surface lamina, 3- vertical clefts to the calcified cartilage extending to <25% of the articular surface, 4- vertical clefts to the calcified cartilage extending to 25–50% of the articular surface, 5- vertical clefts to the calcified cartilage extending to >50–75% of the articular surface, and 6- vertical clefts to the calcified cartilage extending to >75% of the articular surface [18]. Synovitis and osteophytosis were also scored as previously described [19].

For the rat models, knee joints were collected post-mortem at 4 weeks (n = 16), 8 weeks (n = 16), 12 weeks (n = 16), and 16 weeks (n = 20) post-surgery. The joints were fixed in 10% neutral buffered formalin for 48 hours before being decalcified in 10% EDTA for 5 weeks. The joints were split along the coronal plane before being embedded in paraffin wax. Coronal sections (5μm thickness) were taken from the anterior portion of the joint at ~200μm intervals before being stained with H&E. Three stained sections were then scored for chondropathy, synovitis, and osteophytosis using a previously published scoring system [12]. Each study was evaluated by one independent evaluator. The observers were blinded to treatment for all histopathological scoring. The average score for each metric across the 3 sections was reported for each animal.

### Spinal cord immunohistochemistry

Lumbar spinal cords were collected from the traditional and mDMM model at 16 weeks post-surgery. Mice were overdosed with sodium pentobarbital and transcardially perfused with 0.9% saline followed by 4% PFA. Following dissection, spinal cords were post-fixed in 4% PFA for 48 hours before being placed in 30% sucrose in 0.1M phosphate buffer/0.02% sodium azide solution at 4°C. Sections from the lumbar section of the spinal cord (n = 6 sections per mouse, 40μm thickness) from both traditional and mDMM, and sham operated mice were stained for the microglia marker ionized calcium binding adaptor molecule 1 (Iba-1) and the astrocyte marker glial fibrillary acidic protein (GFAP). Sections were blocked with 3% goat serum and 0.3% Triton X-100 in 0.1M PBS for 1 hour at RT. Sections were then incubated with either anti-Iba-1 (Wako, Japan: 019–19741) diluted 1:1000 in Trizma Triton x-100 buffered saline (TTBS) at 4°C for 72 hours or anti-GFAP (Abcam: ab48050) diluted 1:100 in TTBS for 18 hours at RT. Immunolabelling was developed through incubation with either 1:300 Alexafluor 488 conjugated anti-rabbit secondary antibody (Iba-1) or 1:300 Alexafluor 568 conjugated anti-rabbit secondary antibody (GFAP) for 2 hours at RT.

Images of IBA1 and GFAP stained sections of the superficial laminae of the dorsal horn (S2 Fig) were captured using a 20 x 0.4 NA objective lens on a Leica DMIRE2 microscope running micromanager equipped with a Hamamatsu Orca C4642-95 camera. Images were acquired using an exposure time of 150ms (Iba-1) or 50ms (GFAP). Total numbers of activated microglia expressing Iba-1 were counted manually in quadrants from the ipsilateral and contralateral superficial dorsal horn as previously described with microglia being considered activated if the diameter of the cell body was twice the size of the processes [20]. Images of GFAP immunofluorescence were analysed using Velocity v5.5 as previously described and the mean fluorescence grey intensity was determined for each image [20]. The experimenter was blinded to the study conditions for each image during analysis.

### Data analysis

Data were analysed and graphically presented using Prism V.7 (GraphPad, San Diego, California, US). Data were tested for normal distribution using the D’Agostino and Pearson normality test, if data were normally distributed then parametric analyses were used. If data were not normally distributed non-parametric analyses were used. Differences between cartilage damage, synovitis, and osteophytosis between mice with DMM induced pathology and sham operated animals were analysed by Mann Whitney U Test. Spearman’s ρ was used to determine if there was a statistical dependence between weight -bearing asymmetry and joint pathology. Change in weight-bearing asymmetry over time between groups was analysed by 2-way ANOVA with Bonferroni corrected multiple comparisons. Change in log transformed hind paw withdrawal thresholds over time between groups was also analysed by 2-way ANOVA with Bonferroni corrected multiple comparisons. Differences in chondropathy, synovitis, and osteophytosis in MMNX rats compared to sham rats over time was analysed by Mann Whitney U Test. Changes in the number of activated microglia in the ipsilateral dorsal horn were normalised to the number of activated of microglia in the contralateral dorsal horn, and differences between the DMM operated and sham operated mice in both the ipsilateral and contralateral sides were analysed by one-way ANOVA with Tukey’s corrected multiple comparisons. Changes in the GFAP immunofluorescence between DMM and sham mice in both the ipsilateral and contralateral sides were analysed by one-way ANOVA with Tukey’s corrected multiple corrections.

## Results

### Modification of the DMM model alters pain behavior but not joint damage

**Fig 1:**
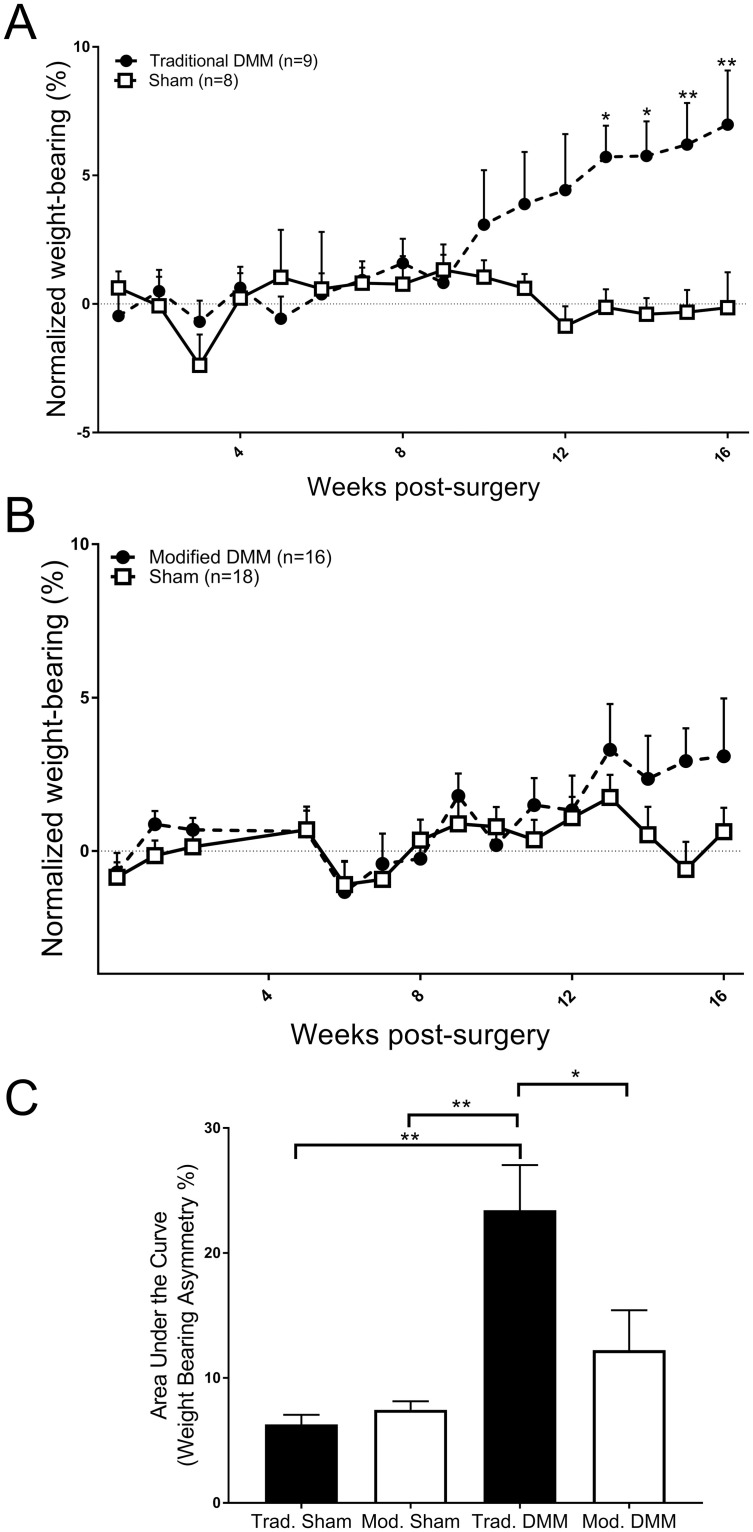
Pain behaviour in the traditional versus modified DMM model of OA. Adult male C57BL/6 mice underwent either traditional destabilisation of the medial meniscus (DMM) (n = 9) or sham surgery (n = 8) (**A**) and weight bearing asymmetry was measured up to 16 weeks post model induction. Adult male C57BL/6 mice underwent either modified destabilisation of the medial meniscus (DMM) (n = 16) or sham surgery (n = 18) (**B**) and weight bearing asymmetry was measured up to 16 weeks post model induction. Data analysed by 2-way ANOVA with Bonferroni corrected multiple corrections. * = p<0.05, ** = p<0.01 DMM vs Sham. Differences in the area under the curve of weight-bearing asymmetry 12 to 16 weeks following traditional or modified DMM surgeries (**C**) was analysed by Kruskal-Wallis test with Dunn’s multiple comparisons. * = p<0.05, ** = p<0.01 when compared to traditional DMM.

In the traditional DMM model there was a slowly progressing increase in weight-bearing asymmetry, compared to sham operated mice (Fig 1A). At week 10 there was a non-significant difference in weight-bearing asymmetry in the DMM mice, compared to sham controls. By week 13 this difference in weight-bearing asymmetry in the DMM group was significant compared to the sham controls. By contrast, following the modified DMM surgery in mice there was no significant change in weight-bearing over 16 weeks, compared to the sham operated mice (Fig 1B). Area under the curve of the analysis from 12 weeks post-surgery through to week 16 shows a significant increase in weight-bearing asymmetry in the traditional DMM model over these time-points when compared to the modified surgery (Fig 1C).

**Fig 2:**
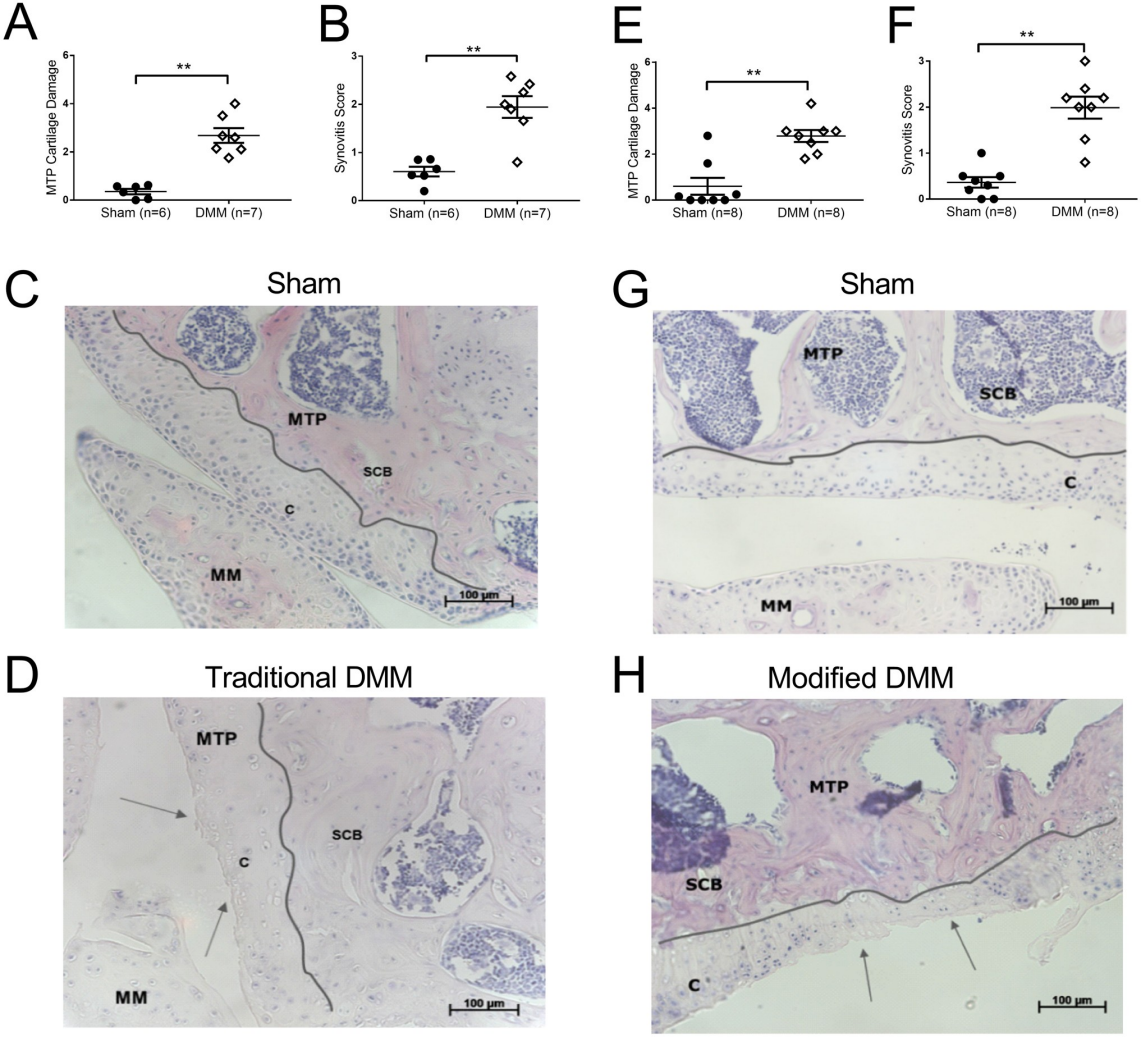
Pathology in the traditional versus modified DMM model of OA. Adult male C57BL/6 mice underwent either traditional destabilisation of the medial meniscus (DMM) (n = 7) or sham surgery (n = 6) (**A, B, C, D**). Cartilage damage (**A**) and synovitis (**B**) were analysed 16 weeks post-surgery. Representative images of sham (**C**) and traditional DMM (**D**) joints. Adult male C57BL/6 mice underwent either modified destabilisation of the medial meniscus (DMM) (n = 8) or sham surgery (n = 8) (**E, F, G, H**). Cartilage damage (**E**) and synovitis (**F**) were analysed 16 weeks post-surgery. Representative images of sham (**G**) and modified DMM (**H**) joints. Data analysed by Mann Whitney U-Test ** = p<0.01 DMM vs Sham.

**Fig 3:**
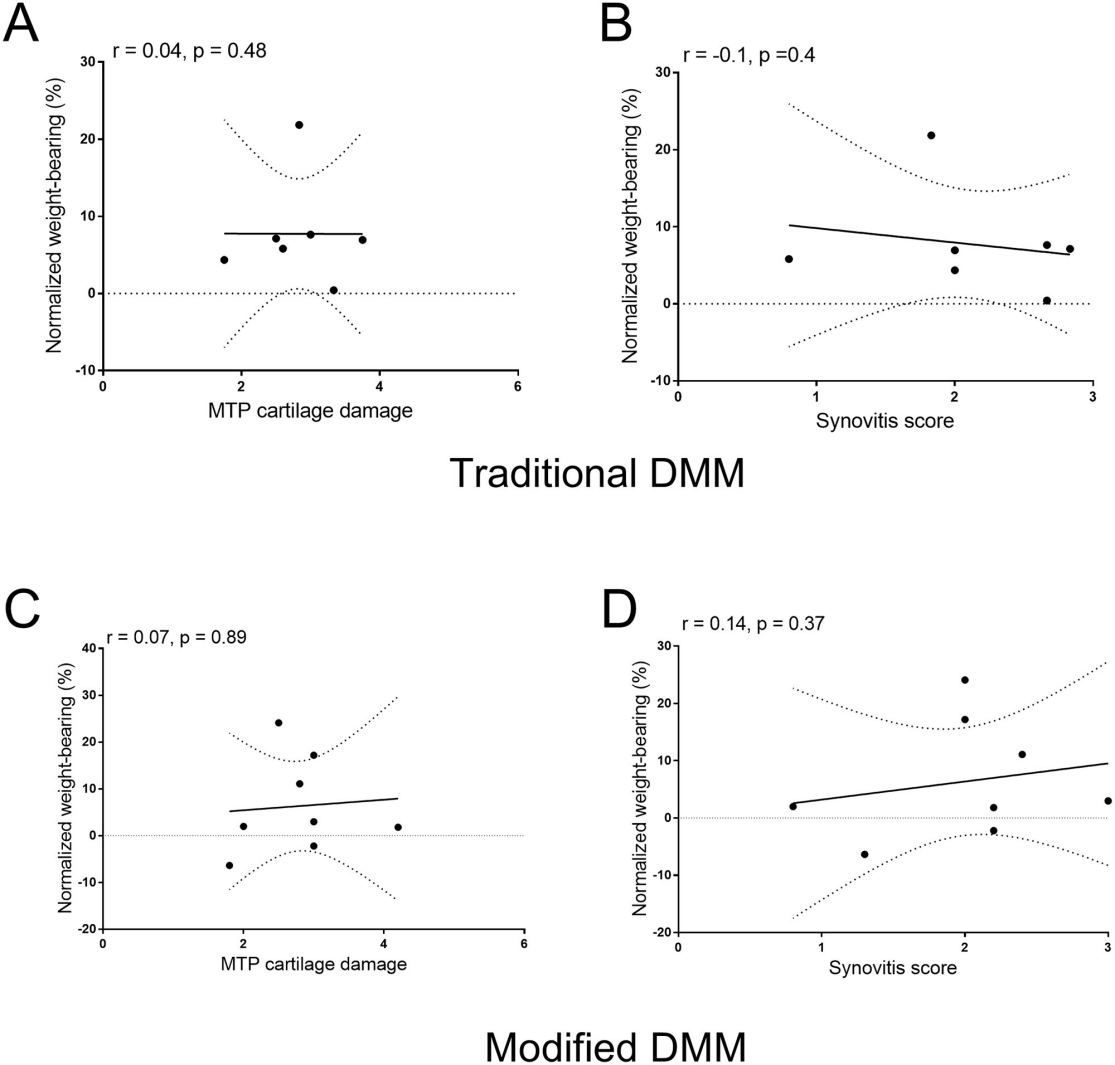
Pain behaviour and joint pathology are not correlated in the traditional or modified DMM models in the mouse. Adult male C57BL/6 mice underwent traditional destabilisation of the medial meniscus (DMM) (n = 9) (**A,B**). Weight bearing asymmetry at 16 weeks post model induction was not correlated with either cartilage damage (**A**) or synovitis (**B**). Adult male C57BL/6 mice underwent modified DMM (n = 16) (**C,D**). Weight bearing asymmetry at 16 weeks post-modified DMM was not correlated with either cartilage damage (**C**) and synovitis (**D**). Data analysed Spearman’s rho.

At 16 weeks after the induction of the traditional DMM model, ipsilateral knee joints had chondropathy in the medial tibial plateau (Fig 2A) as well as increased synovitis (Fig 2B), compared to sham operated mice (Fig 2C and 2D). There was also a significant, but small, increase in osteophytosis in knee joints from DMM mice, compared to sham operated mice at this timepoint (Sham: 0.02±0.01, DMM: 0.22±0.05). At 16 weeks after induction of the modification of DMM surgery, ipsilateral knee joints also had chondropathy of the medial tibial plateau, which was significant compared to the sham control (Fig 2E). Likewise, there was significant synovitis in the modified DMM group, compared to sham operated mice (Fig 2F). However, there was no evidence for osteophytosis in any of the knee joint sections from the modified DMM or sham control surgery (Sham: 0±0, DMM: 0±0). Overall, synovitis and chondropathy at 16 weeks post-surgery was comparable between the two variations of the DMM model induction despite the differences in pain behaviour between the two models. There were no significant correlations between pain behaviour and joint pathology for either the traditional or modified DMM surgery (Fig 3).

**Fig 4:**
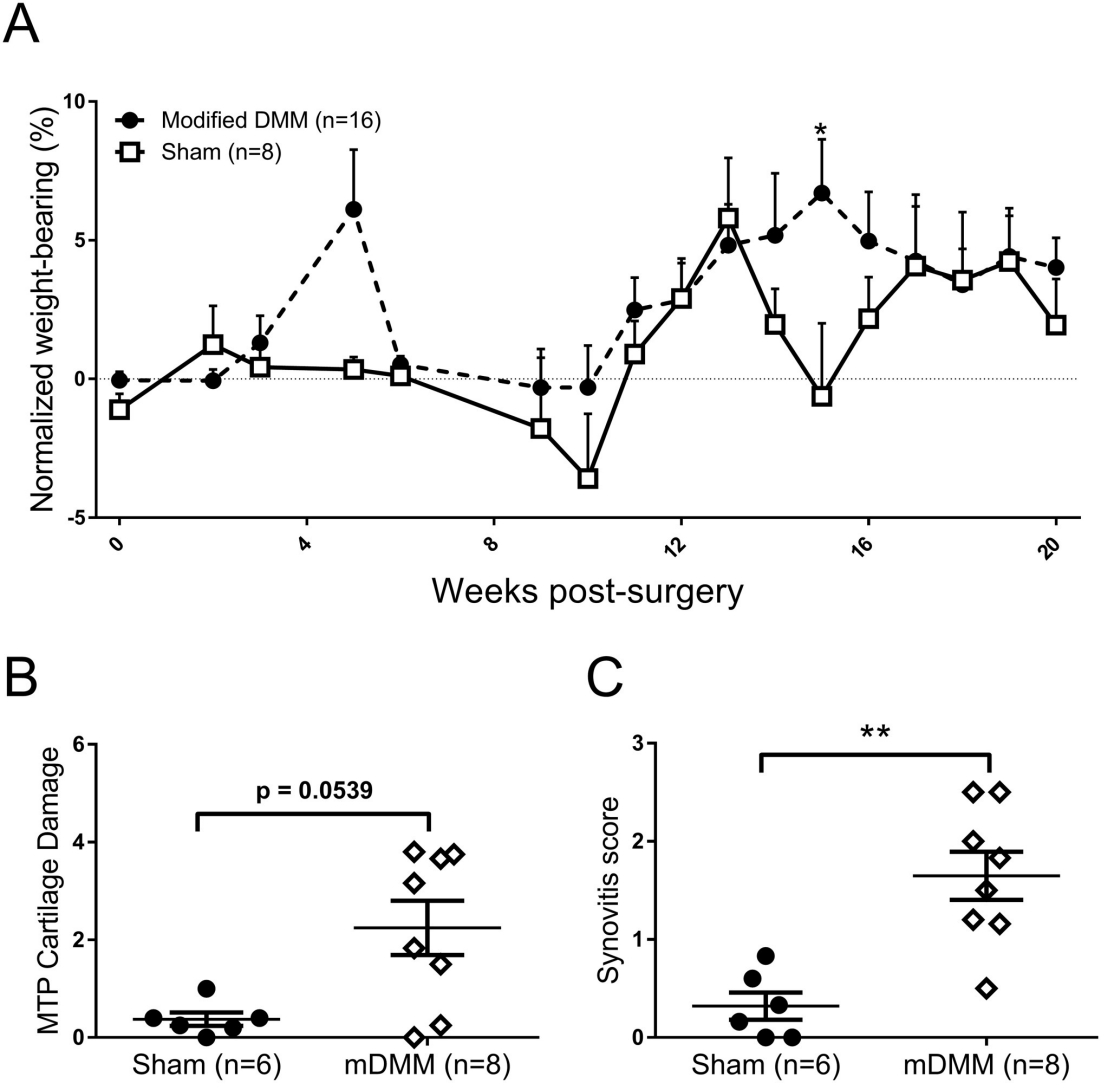
No effect of modified DMM surgery on pain behavior up to 20 weeks post-surgery. **(A)** Adult male C57BL/6 mice underwent either modified destabilisation of the medial meniscus (DMM) (n = 16) or sham surgery (n = 8) and weight bearing asymmetry was measured up to 20 weeks post model induction. Data analysed by 2-way ANOVA with Bonferroni corrected multiple corrections. * = p<0.05. (**B**) Cartilage damage and (**C**) synovitis were analysed 20 weeks post-surgery. Data analysed by Mann Whitney U Test, ** = p<0.01 DMM vs Sham.

We next considered that modification of the DMM model may have a delayed the onset of a pain phenotype, which was tested in a second cohort of mice studied until 20 weeks following modified DMM surgery. However, at least until this timepoint, weight-bearing between the hind paws remained unaltered in the modified DMM mice and was comparable to the sham control at this timepoint (Fig 4A). Importantly, chondropathy and synovitis scores were still increased at 20 weeks in the modified DMM group compared to the sham controls (Fig 4B and 4C).

**Fig 5:**
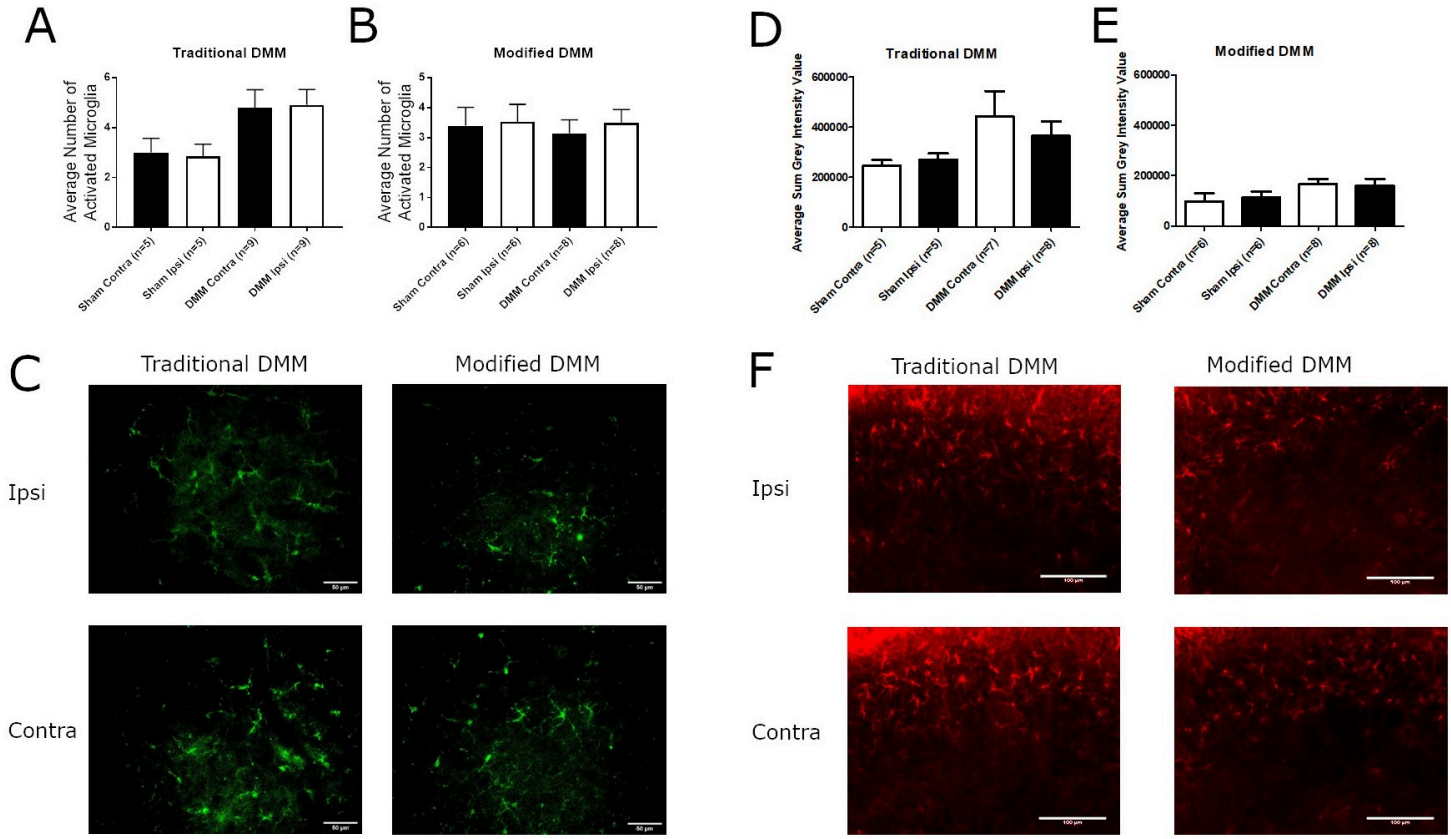
Quantification of neuroimmune cell activation in the dorsal horn of the spinal cord. Adult male C57BL/6 mice underwent traditional DMM, modified DMM, or sham surgery. Dorsal horn spinal cord Iba-1 immunostaining for microglia and GFAP immunofluorescence for astrocytes was quantified at 16 weeks after surgery. (**A**) Quantification of Iba-1 immunostaining in mice that underwent traditional DMM (n = 8) or sham surgery (n = 6). (**B**) Quantification of IBA1 immunostaining in mice that underwent modified DMM (n = 8) or sham surgery (n = 6). Data are reported as average number of activated microglia in the ipsilateral and contralateral dorsal horn. (**C**) Representative images of Iba-1 staining in the dorsal horn of the spinal cord. Data were analysed by Kruskal-Wallis test with Dunn’s multiple comparisons, no significant differences observed. GFAP immunofluorescence data are the average sum grey intensity value measured using Velocity software. (**D**) Quantification of GFAP immunostaining in mice that underwent traditional DMM (n = 8) or sham surgery (n = 6). (**E**) Quantification of GFAP immunostaining in mice that underwent modified DMM (n = 8) or sham surgery (n = 6). (**F**) Representative images of GFAP immunostaining in the superficial dorsal horn of mice that underwent traditional or modified DMM surgery. Data were analysed by one-way ANOVA with a Dunnett’s multiple comparison, no significant differences observed.

It was apparent that the modification of the surgical induction of the DMM resulted in differing pain responses despite presenting with a similar cartilage damage and synovitis. Changes in the glial cell response in the dorsal horn of the spinal cord have previously been linked to the chronification of pain in the monosodium iodoacetate (MIA) model of OA in the rat [20]. We undertook immunohistochemistry for IBA1 (a marker for microglia) and GFAP (a marker for astrocytes) to investigate if there were any differences in the glial response in the dorsal horn of the spinal cord between the variants of the DMM surgery. At the timepoints studied there were no significant differences in microglia activation (Fig 5A and 5B) or in astrocyte immunoreactivity (Fig 5C and 5D) between sham or DMM animals in either of the surgical conditions at 16 weeks post-surgery.

### Pain behavior associated with modified MNX surgery in the rat

**Fig 6:**
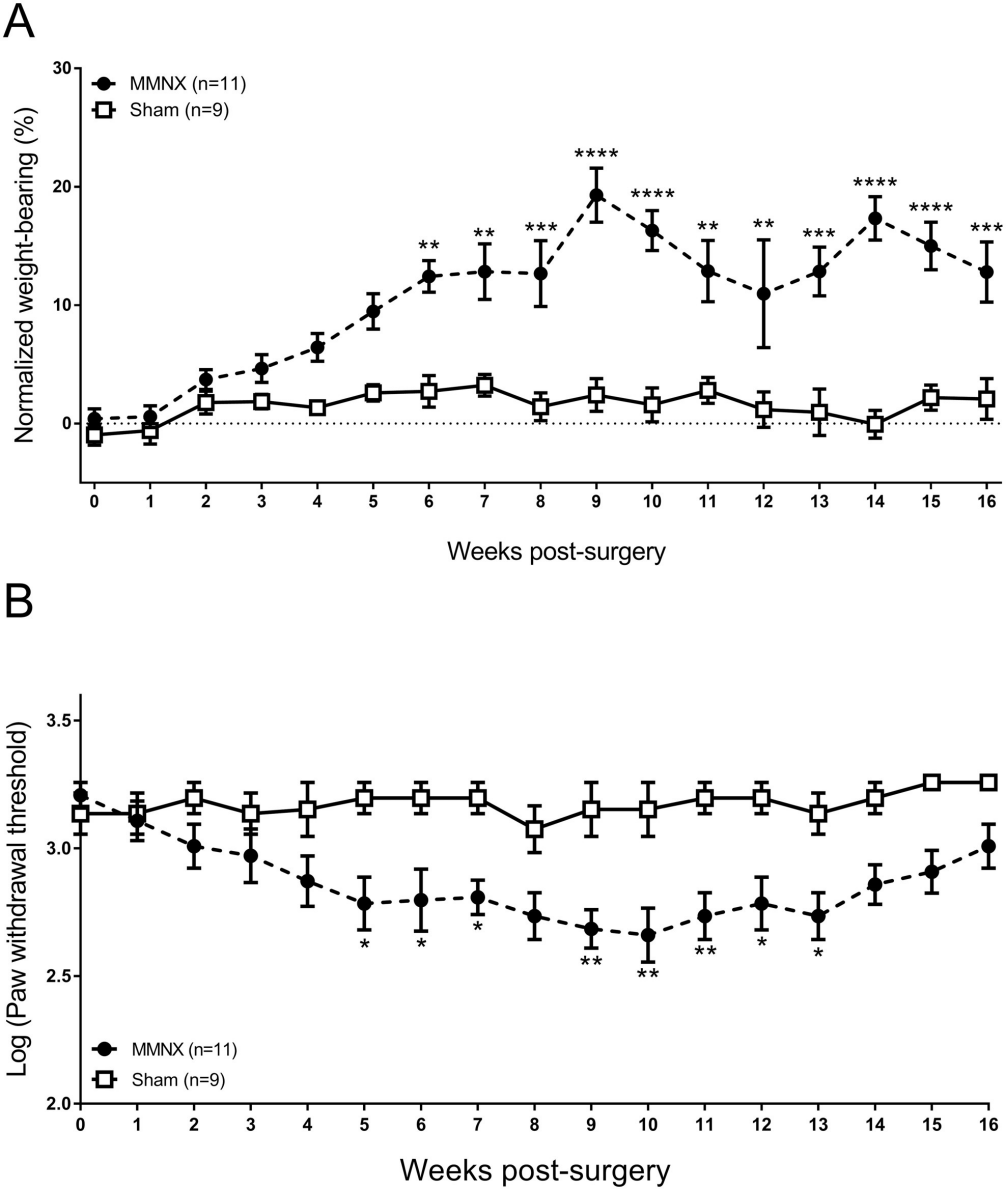
Pain behaviour in the modified MNX model of OA in the rat. Adult Sprague Dawley Rats underwent either modified MNX surgery (n = 11) or sham surgery (n = 9). Weight bearing asymmetry (**A**) and paw withdrawal thresholds (**B**) was measured up to 16 weeks post model induction. Data analysed by 2-way ANOVA with Bonferroni corrected multiple corrections. * = p<0.05, ** = p<0.01, *** = p<0.001, **** = p<0.0001 MMNX vs Sham.

We hypothesised that refining the MNX surgery in the rat to more closely resemble the DMM surgery in the mouse would result in a slowly progressing pain phenotype. Following modification of the MNX surgery, weight-bearing asymmetry increased slowly over time with significant differences evident at 6 weeks post-surgery compared to sham controls (Fig 6A). This increase in weight-bearing asymmetry persisted until the end of the study at 16 weeks post-surgery. A second measure of pain behavior, absolute ipsilateral hind paw withdrawal thresholds were reduced (more sensitive) in the modified MNX model. A significant decrease in paw withdrawal thresholds in the modified MNX model was evident from 5 weeks post-surgery, compared to sham controls (Fig 6B). Contralateral hind paw withdrawal thresholds were not altered in the modified MNX model, compared to baseline or sham controls (S3 Fig).

**Fig 7:**
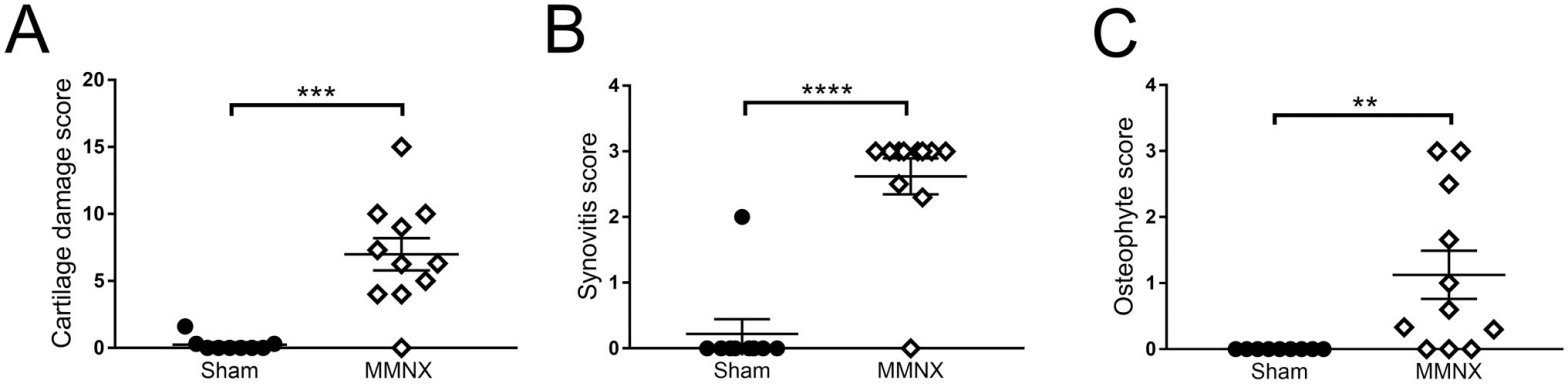
Joint pathology changes 16 weeks following induction of the modified MNX rat model of OA pain. Adult male Sprague Dawley rats underwent either modified MNX or sham surgery. Cartilage damage (**A**), synovitis (**B**), and osteophytes (**C**) were analysed 16 weeks post-surgery. Data analysed by Mann Whitney U test. ** = p<0.01, *** = p<0.001, **** = p<0.0001 MMNX vs Sham.

At 16 weeks following modified MNX surgery there was significant chondropathy compared to sham controls (Fig 7A). Similarly, both synovitis (Fig 7B) and osteophytosis (Fig 7C) were significantly increased compared to sham controls at 16 weeks. As was found in the DMM models, there were no significant correlations between pain behaviour and features of joint pathology at 16 weeks post-surgery (S4 Fig).

### Onset of joint pathology in the modified MNX model

**Fig 8:**
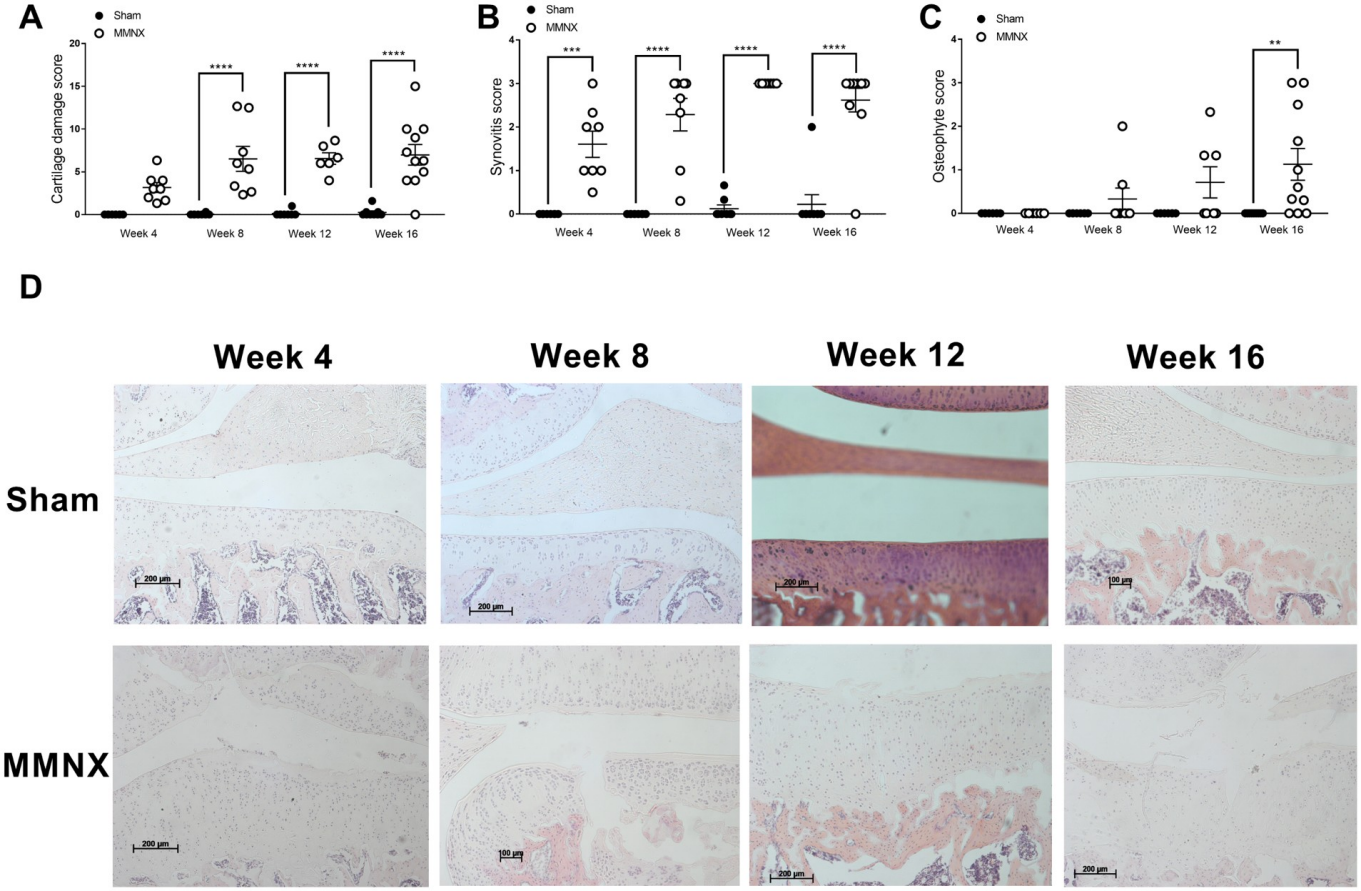
Time-course of joint pathology changes in the modified MNX model in the rat. Adult male Sprague Dawley rats underwent either modified MNX or sham surgery. Cartilage damage (**A**), synovitis (**B**), and osteophytes (**C**) were analysed 4 (n = 8 per group), 8 (n = 8 per group), 12 (n = 8 per group) and 16 (n = 8 per group) weeks post-surgery. Data analysed by one-way ANOVA with Bonferroni’s corrected multiple comparisons ** = p<0.01, *** = p<0.001, **** = p<0.0001 MMNX vs Sham. Representative images (20x) of the medial tibial plateau highlight the progression of cartilage damage in this model (**D**).

To understand how the slowly developing pain behavior from 5 weeks following MMNX surgery potentially relates to the time course of joint pathology we undertook a more comprehensive time-course of joint pathology in this model. Joint pathology was quantified at 4, 8, 12, and 16 weeks post MMNX surgery, mapping onto the timepoints prior to changes in pain behavior and once it was evident. The earliest feature of joint pathology was synovitis at 4 weeks following MMNX surgery (Fig 8A). Significant cartilage damage was evident at 8 weeks post-surgery (Fig 8B) and osteophyte score was only significantly increased in the MMNX model at the final 16-week timepoint (Fig 8C). Representative images of the medial tibial plateau are shown in Fig 8D.

## Discussion

The aim of this study was to investigate how refining the surgical induction of established small rodent models of OA influenced pain and joint pathology phenotypes. Modification of the DMM surgery in the mouse resulted in an absence of significant weight bearing asymmetry despite the knee joints displaying similar chondropathy and synovitis to the traditional DMM model. Modification of the MNX surgery in the rat resulted in a slowly progressing model of OA, in which synovitis was evident prior to the onset of pain behaviour and significant chondropathy manifested later.

The time-course of changes in weight bearing asymmetry and the extent of joint pathology following the traditional DMM surgery was consistent with previous studies. Increases in weight-bearing asymmetry were significant compared to sham controls from 13 weeks post-surgery, as previously described [9, 21, 22]. Mild joint damage has been reported from 4 weeks post DMM surgery, with worsening over time [8, 9, 23, 24]. In the traditional DMM model we observed significant chondropathy and synovitis at 16 weeks post model induction, compared to sham controls. In the modified DMM model, pain behaviour was absent despite synovitis and cartilage damage of the joint, which was comparable to the traditional DMM model. These data illustrate a potential dissociation between cartilage damage, inflammation, and pain, which may be reflective of asymptomatic OA [25]. However, it is important to note that our study design investigated histological damage at the end point of the rodent models and therefore correlation analysis of these data are limited in their power. Previous work in a rat model of combined MCL and medial meniscus transection found limited associations between pain behavioural surrogates and pathological changes which supports our data [26].

Understanding how differences in the induction of the two versions of DMM surgery relate to the manifestation of pain behaviour may provide insight to the underlying drivers of OA pain. The modified DMM surgery left the MMTL intact whereas it was fully transected in the traditional surgery. Previous work has shown that the complete removal of the meniscus resulted in a reduced pain phenotype and pathology compared to transection [27]. Therefore, it is possible that the greater damage to the meniscus in the traditional DMM model makes an important contribution to pain behaviour. Clinically it has been shown that menisci from patients with OA are more highly vascularised and innervated than those without chondropathy [28], separately meniscal tears and patient reported pain have been shown to be associated [29]. The absence of pain in the presence of pathology in the modified DMM highlights that pathology may develop asymptomatically in this model. Radiographic OA in humans is not necessarily associated with joint pain [4], and asymptomatic joints even in people with RA may display synovitis [30]. It has been hypothesised that increased nociceptive innervation may drive some pathological changes. Previous work has shown that desensitisation of TRPV1 positive neurons significantly reduced MIA induced bone erosion [31] with sensory neurons contributing to the regulation of bone resorption [32]. Our data suggest that whilst sensory innervation may have a role in mediating bone damage, pain itself isn’t prerequisite for synovitis and cartilage damage.

The roles of spinal excitability and central sensitization in OA pain have been widely described in animal models [16, 33] and patient studies [34]. To investigate whether the different pain outcomes in the two variations of the DMM model could be explained by spinal sensitization, we quantified glial cell activation in the dorsal horn of the spinal cord. Spinal glial cell activation has been shown to be important for the development and maintenance of central sensitization in more rapidly developing models of neuropathic and osteoarthritis pain [20, 35, 36]. However, we found no evidence for activation of microglia or astrocytes in the ipsilateral dorsal horn of the spinal cord following traditional or modified DMM surgery at 16 weeks post-surgery, compared to sham controls. Previously, microglia activation was reported bilaterally in the dorsal horn of the spinal cord at 8 weeks post DMM surgery [37], which may suggest that our later time point missed the activation of microglia which is known to be transient in models of both OA and neuropathic pain [20, 38]. Spinal astrocyte activation often follows microglial activation in OA and neuropathic pain states [20, 38]. Given that GFAP IF was also not altered in the dorsal horn of the spinal cord, this suggests that astrocyte activation at this level is not overtly associated with weight-bearing asymmetry in the DMM model at the 16-week timepoint. An investigation into changes in distal pain behaviour, as measured by hind-paw withdrawal thresholds, might provide more insight into potential changes in central sensitization between the two models. Previous work has shown greater nociceptive innervation in knee joint in the DMM model at 16 weeks [39], whether this is also evident in the modified DMM study is unknown. Due to the availability of tissues from these mice, this question was beyond the scope of our study. Whether changes in knee innervation lead to altered responses of spinal neurones in the DMM model is also unknown.

To further investigate how features of joint pathology map to pain behaviour, we refined the MNX model of OA in the rat to produce a slow progressing model of OA. Following modification of the MNX model, rats exhibited significant pain on loading of the joint (measured by weight-bearing asymmetry) from 6 weeks post-surgery, much later than normally evident following MNX surgery which is generally significant from as early as 3 days post-surgery [40, 41]. In addition, hind paw withdrawal thresholds were significantly lowered from 5 weeks after modified MNX surgery, again much later than that seen in the MNX model [16, 42]. Synovitis was evident at 4 weeks following modified MNX surgery, preceding pain behaviour. By contrast, significant cartilage damage was evident from 8 weeks post-surgery, after the onset of pain behaviour. Clinically, synovitis and synovial thickness are both associated with pain in people with OA [43, 44], our data suggest that synovitis may be an important pathological feature that precedes the onset of chronic joint pain.

In both the traditional DMM and modified MNX models there was evidence of significant osteophytosis 16 weeks post–surgery, well beyond the manifestation of pain behaviour. It is important to note that osteophytosis is an end stage outcome of bone remodelling in OA [45], and features of early bone-remodelling were not the focus of this study. Our data support the dissociation between clinically diagnosed OA, using joint space narrowing and osteophytosis, and pain [4]. However early subchondral remodelling events could be important drivers of OA pain with previous research highlighting the analgesic potential of the bisphosphonates in rats and humans [46, 47].

The delayed onset of pain behaviour as well as its persistence up to 16 weeks following modified MNX surgery is of particular benefit for investigating the mechanisms underlying early OA pain, its chronification and the relationship with joint pathology. Our data add to the literature on the use of slow progressing models of OA in the rat. Effects of DMM surgery (in the rat) or anterior cruciate ligament transection with partial medial meniscectomy (ALCX) on joint pathology have been reported. Both cartilage damage and subchondral bone defects were evident at 1 week post-surgery in the rat DMM model [14], and decreased bone marrow density was evident 1 month post-ALCX [48]. However, there is limited information on the progression of pain behaviour in these slower developing models of OA. A pilot study in a small cohort of female rats found no significant differences in pain outcomes between DMM rats and sham controls over the 42 days post-surgery [49]. Work in an MCL transection model has shown the onset of significant joint pathology over the course of 6 weeks, with a transient change in gait compensation seen in hind paw withdrawal thresholds [26, 50]. Our data in the mice suggests that DMM induced chronic weight-bearing asymmetry may occur later than this timepoint, and significant weight-bearing differences in the modified MNX model in the rat were seen from 42 days post-surgery. Previous work in the rat DMM model reported an increase in MMP13 expression in the cartilage and subchondral bone [14], which is known to activate TNFα [51]. These data also support an early inflammatory phenotype in these surgical models.

Greater understanding of how structural changes in the joint may mediate chronic pain in slowly progressing models of OA could improve the translation of findings from the laboratory. Our data highlight how variation in model induction can have important effects on the endpoints of experiments. These results support a recent editorial which commented on the variety of outcomes seen in studies utilizing the DMM model, and the need for more specific detail when reporting data that uses these surgical models [52]. The differences between joint pathology and pain behaviour in these pre-clinical models mirrors the complex relationships between these different parameters in human OA.

## Supporting information

S1 Fig Photographs of the modified DMM model of OA in the mouse. This series of images shows the surgical procedure for the modified DMM. **A**: The knee prepared for surgery. **B**: The location of the medial collateral ligament (MCL), medial meniscus (MM), and infrapatellar ligament (IPL). **C**: The blunt dissection of the connective tissues on top of the medial meniscus, after this step the sham surgery is complete. **D**: The blue line indicated the transection of the ligaments attaching the MM to the medial tibial plateau. **E**: The end point of the surgery whereby the medial meniscus is destabilised. **F**: Following surgeries the wound was sealed with wound clips.
(TIF)

S2 Fig Schematic of the spinal cord region of interest used for immunohistochemistry. The red box illustrates the region of interest imaged and quantified for the immunohistochemistry. Images were taken from both the ipsilateral and contralateral side of the spinal cord.
(TIF)

S3 Fig Contralateral hind paw withdrawal thresholds in the modified MNX model in the rat. Adult Sprague Dawley Rats underwent either modified MNX (n = 11) or sham surgery (n = 9). Contralateral paw withdrawal thresholds were measured up to 16 weeks post-surgery. Data were analysed by 2-way ANOVA with Bonferroni corrected multiple corrections. No significant differences between groups was observed.
(TIF)

S4 Fig Pain behaviour and joint pathology are not correlated in the modified MNX model in the rat. Adult Sprague Dawley Rats underwent modified meniscal transection surgery (n = 11) and weight-bearing asymmetry was correlated with cartilage damage (**A**), synovitis (**B**), and osteophyte score (**C**). Data analysed Spearman’s rho.
(TIF)

S1 Data (XLSX)
