# Supplementary figures and images for: Refining surgical models of osteoarthritis in mice and rats alters pain phenotype but not joint pathology
Source: PLoS One. 2020 Sep 29;15(9):e0239663. doi: 10.1371/journal.pone.0239663 (PMC7523978; doi:10.1371/journal.pone.0239663)

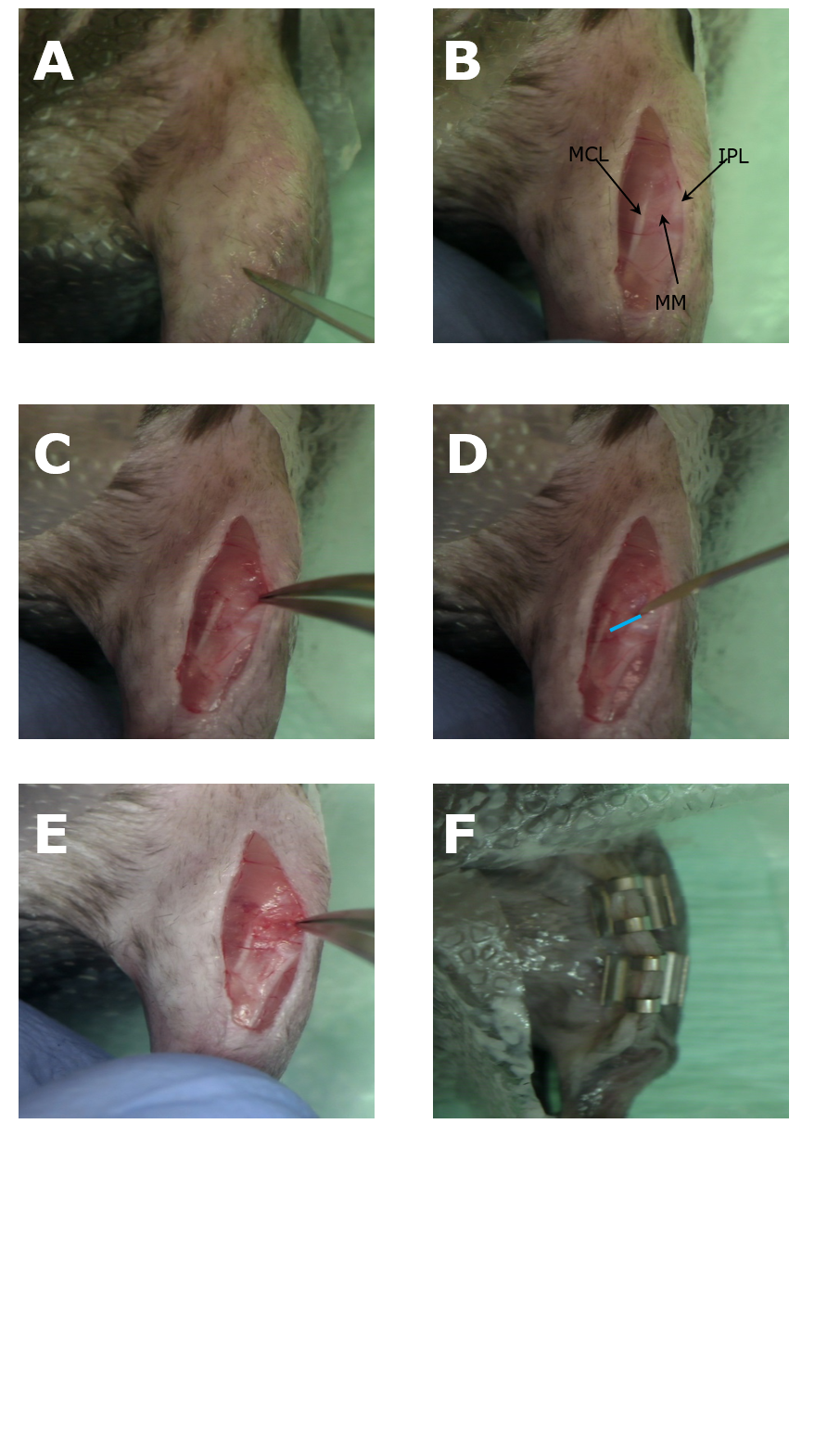

Supplement: S1 Fig — This series of images shows the surgical procedure for the modified DMM. A: The knee prepared for surgery. B: The location of the medial collateral ligament (MCL), medial meniscus (MM), and infrapatellar ligament (IPL). C: The blunt dissection of the connective tissues on top of the medial meniscus, after this step the sham surgery is complete. D: The blue line indicated the transection of the ligaments attaching the MM to the medial tibial plateau. E: The end point of the surgery whereby the medial meniscus is destabilised. F: Following surgeries the wound was sealed with wound clips. (TIF) [file pone.0239663.s001.tif]

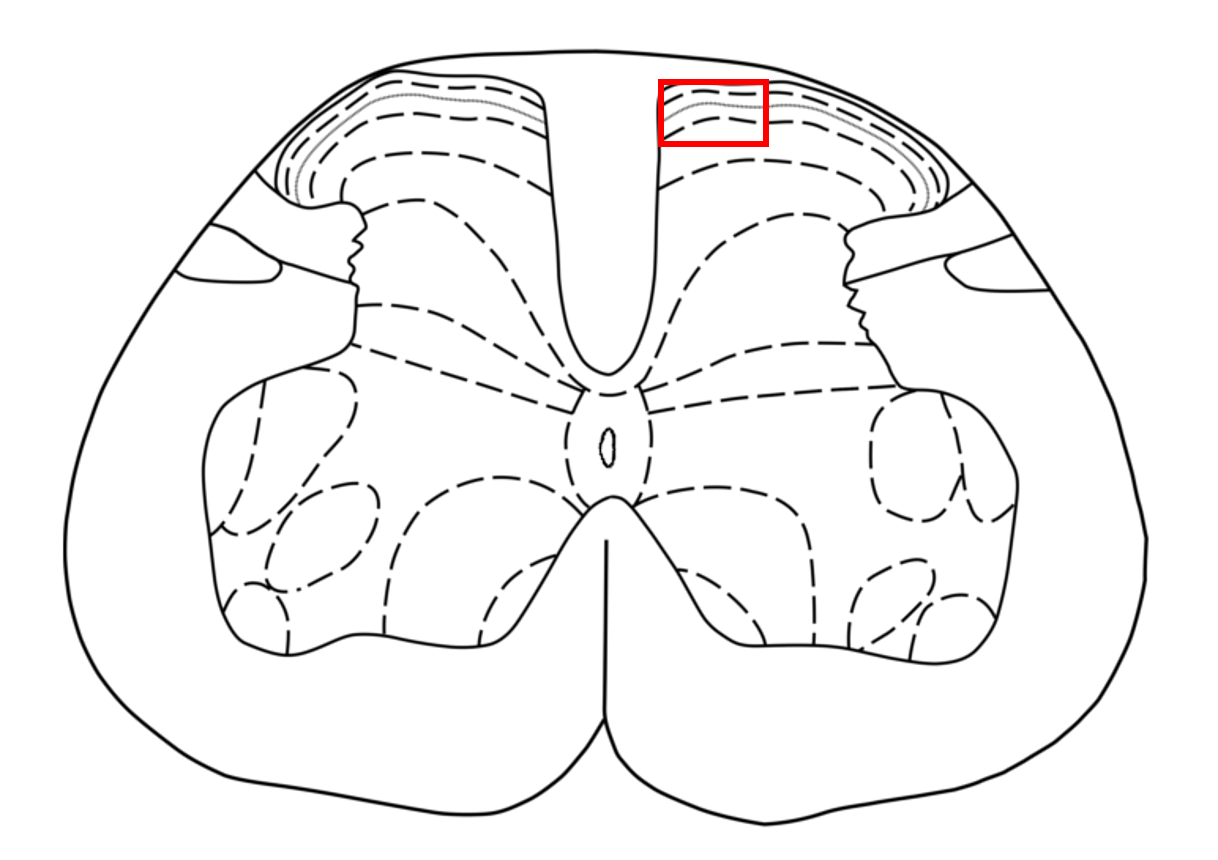

Supplement: S2 Fig — The red box illustrates the region of interest imaged and quantified for the immunohistochemistry. Images were taken from both the ipsilateral and contralateral side of the spinal cord. (TIF) [file pone.0239663.s002.tif]

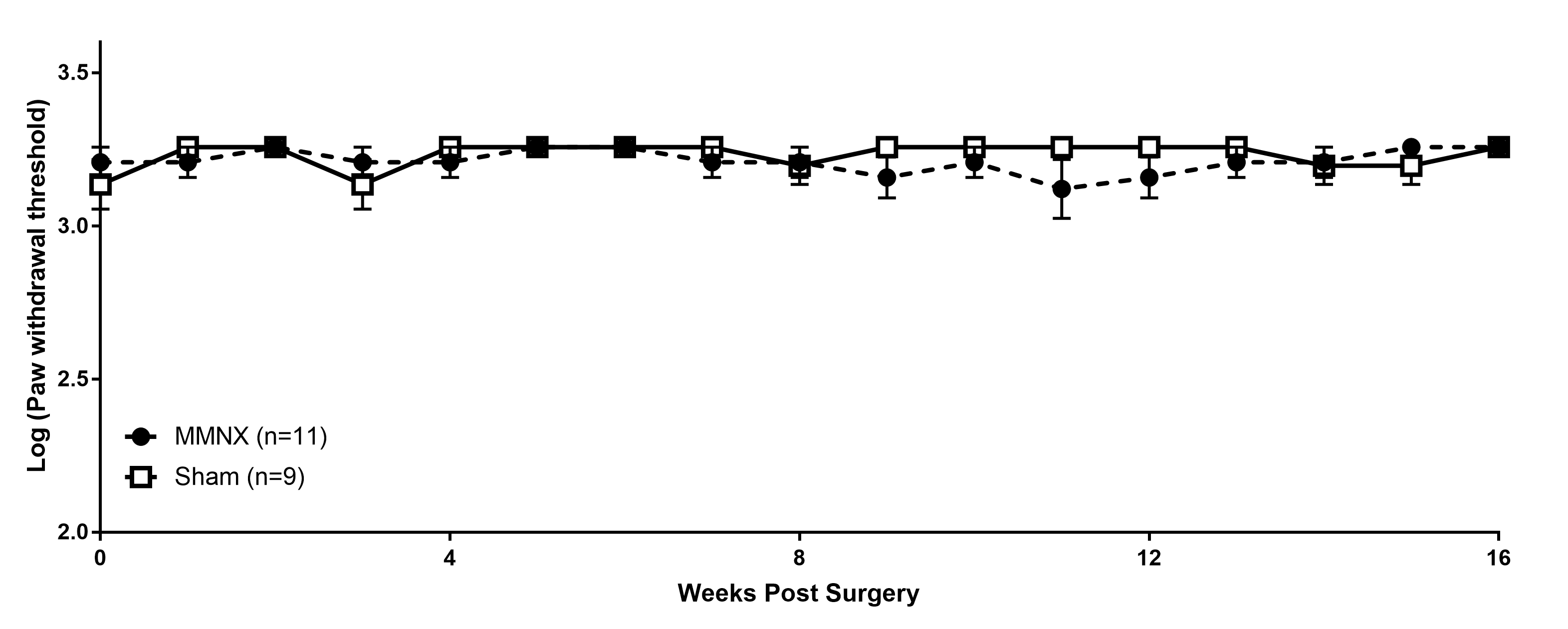

Supplement: S3 Fig — Adult Sprague Dawley Rats underwent either modified MNX (n = 11) or sham surgery (n = 9). Contralateral paw withdrawal thresholds were measured up to 16 weeks post-surgery. Data were analysed by 2-way ANOVA with Bonferroni corrected multiple corrections. No significant differences between groups was observed. (TIF) [file pone.0239663.s003.tif]

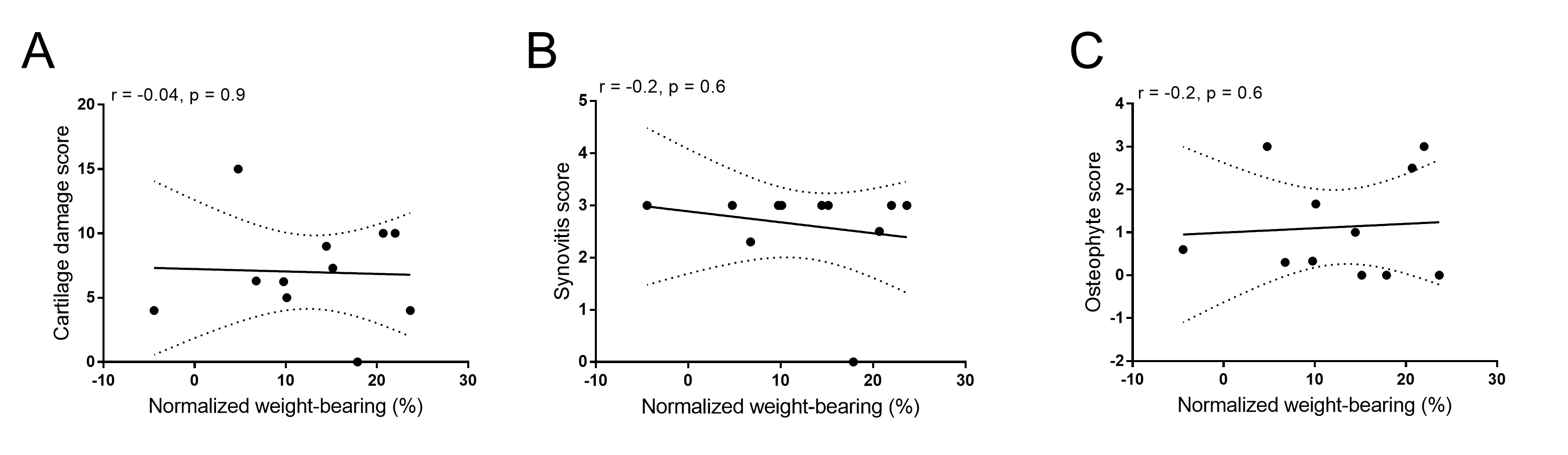

Supplement: S4 Fig — Adult Sprague Dawley Rats underwent modified meniscal transection surgery (n = 11) and weight-bearing asymmetry was correlated with cartilage damage (A), synovitis (B), and osteophyte score (C). Data analysed Spearman’s rho. (TIF) [file pone.0239663.s004.tif]
